# Dr. John William Dowlings

**Published:** 1914-07

**Authors:** 


Dr. John William Dowling, N. Y. Homoeopathic, 1886, died in Middletown, May 11, of cerebral hemorrhage, aged 49. He was Prof. of Medicine and Secretary of the college at which he graduated.

